# Spontaneous Emergence of Homochiral Suspensions from Racemic Solutions via Stochastic Nucleation

**DOI:** 10.1021/jacs.5c02651

**Published:** 2025-05-25

**Authors:** Leif-Thore Deck, Mercedeh Sadat Hosseinalipour, Marco Mazzotti

**Affiliations:** † 111951 Institute of Energy and Process Engineering, ETH Zurich, Zurich 8092, Switzerland; ‡ Yusuf Hamied Department of Chemistry, 2152 University of Cambridge, Cambridge CB2 1EW, U.K.

## Abstract

Homochirality represents a hallmark of life, and its emergence on the prebiotic Earth remains elusive. Here, we demonstrate a spontaneous pathway to homochirality, where a conglomerate-forming chiral species (*N*-(2-methylbenzylidene)-phenylglycine amide, NMPA) evolves from an initial perfectly symmetric state, i.e., a racemic solution, to a fully homochiral state, where only enantiopure crystals of one handedness are present in the suspension. It entails first the establishment of supersaturated conditions, e.g., because of either solvent evaporation or cooling, then stochastic nucleation of enantiopure crystals as the symmetry-breaking event, and finally asymmetry amplification and complete deracemization via temperature fluctuations in a racemizing solution (thanks to the presence of the base 1,8- diazabicyclo[5.4.0]­undec-7-en, DBU). After developing a stochastic modeling platform and an experimental setup, we confirm the plausibility of this pathway through both detailed simulations and laboratory experiments. We also show how from a variety of local homochiral states of different handedness a global homochiral state may emerge via merging and deracemizing. Because the external conditions triggering supersaturation creation, nucleation, and deracemization via temperature-cycling must have existed also on the prebiotic Earth, the proposed spontaneous pathway may have plausibly played a role in the emergence of life on Earth.

## Graphical abstract

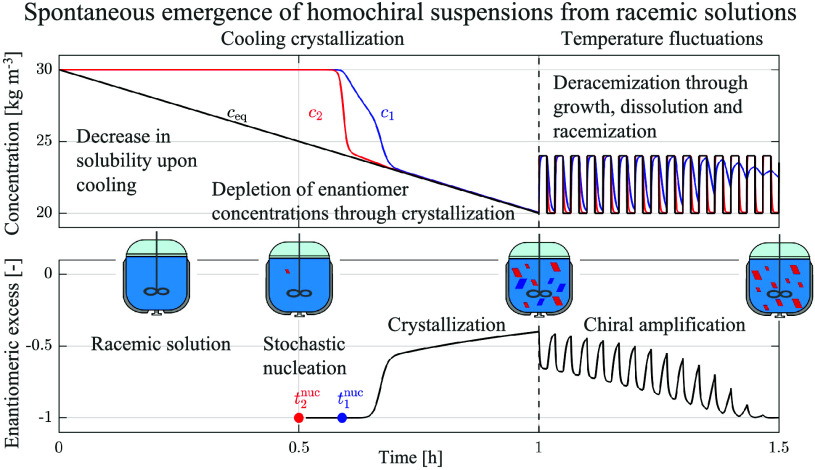

## 1 Introduction

The emergence of the single-handedness of biological molecules on Earth, called homochirality, is considered to have played a key role in the emergence of life.^1,2^ Yet, the fundamental mechanisms behind the transition from racemic to homochiral chemical environments remain largely unclear, as chemical reactions with achiral or racemic starting materials (as available on the prebiotic Earth) generally yield racemic products. In his seminal paper in 1953, Frank^3^ mathematically demonstrated that any pathway to homochirality must comprise first the generation of a (possibly very small) chiral asymmetry and second its amplification until only a single-handedness remains. Numerous phenomena have been studied as potential sources for chiral asymmetries,^4,5^ such as the parity-violating energy difference,^6–8^ the effect of chirality-induced spin selectivity,^9–11^ and the crystallization of conglomerates^12–14^ (compounds where the two enantiomers form distinct enantiopure crystals instead of racemic crystals, which comprise both enantiomers in a regular racemic lattice).

In contrast, only two pathways for chiral amplification have been discovered. Soai et al.^15^ proposed a *chemical pathway* based on enantioselective autocatalysis, i.e., the notion that a chiral reaction product catalyzes its own formation. While the reaction discovered by Soai is of no prebiotic relevance, Deng et al.^16^ have recently observed chiral amplification in prebiotic ligation reactions. Viedma^17^ proposed a *physical pathway* to chiral amplification by demonstrating that under certain conditions, the enantiomeric excess in a suspension of conglomerate crystals increases until homochirality has been achieved. This process is known as Viedma ripening or solid-state deracemization and has been implemented through multiple technical means, namely, isothermal grinding or milling,^17,18^ application of ultrasound,^19,20^ temperature-cycling,^21,22^ solvent-cycling,^23,24^ and mechano-chemistry.^25^ A wide range of chemical species have been shown to undergo deracemization.^26–28^ In this context, we have recently proved that crystalline suspensions of conglomerate-forming species deracemize from an arbitrarily small initial asymmetry, upon arbitrarily small temperature fluctuations in the presence of a racemization reaction in solution, whenever a simple and ubiquitous condition is met, namely that crystal dissolution is faster than crystal growth.^29^

Here, we demonstrate that crystallization naturally creates the required initial asymmetry because the first step in the formation of new crystals, i.e., nucleation, is stochastic. On this basis, we establish a general and prebiotically plausible *single-step* pathway for the transformation of a perfectly racemic solution into a homochiral suspension, building on (i) theoretical considerations, (ii) simulations using a first-principles mathematical model, and (iii) experiments using the chiral compound *N*-(2-methylbenzylidene)-phenylglycine amide (NMPA). Both simulations and experiments confirm the theory.

## 2 Background

### 2.1 Frank Conjectures a Mechanism to Homochirality

In 1953, Frederick C. Frank argued that any process that may generate an asymmetric entity from a symmetric initial state (he explicitly refers to the “production of living molecules” in a prebiotic environment) is by nature a statistically rare event, and cannot as such preserve the initial symmetry; therefore its outcome is asymmetric.^3^ Frank did not specify which physical or chemical mechanisms may be involved in such symmetry-breaking first step though. He also showed that even very simple mathematical models of the interaction between two enantiomers in a well-mixed system may amplify irreversibly any initial asymmetry. He concluded his seminal article maintaining that “spontaneous asymmetric synthesis is a natural property of life” and that “a laboratory demonstration may not be impossible.”

Frank’s model consists of two symmetric ordinary differential equations that describe the evolution of a homogeneous, reacting, batch, binary system, whereby the rate of increase of each species (enantiomer) is the sum of a positive term accounting for autocatalysis and of two negative terms accounting for self-inhibition and for cross-inhibition (see for details the Supporting Information). Whether cross-inhibition is stronger than self-inhibition is controlled by the sign of a single rate constant parameter (called *k*_3_ in the original paper). Although Frank considered only the case where cross-inhibition is equal to or stronger than self-inhibition (*k*_3_ ≥ 0), we broaden the analysis to also consider the case where the opposite occurs. Thus, we can conclude that amplification of the initial asymmetry occurs if and only if cross-inhibition is strictly stronger than self-inhibition, i.e., if and only if *k*_3_ > 0.

Frank also noted that, because of the stochastic nature of the initial symmetry-breaking event, either handedness may have prevailed in various places on the prebiotic Earth. Nevertheless, he considered it obvious that through successive interactions between colonies of the two kinds of handedness, which were statistically not identical, a single-handedness would ultimately survive and dominate the whole planet, i.e., worldwide homochirality would develop.

### 2.2 Nucleation is Stochastic

The stochastic nature of primary nucleation is well established; it has been observed for different types of nucleation,^30,31^ that is, for both homogeneous (nuclei are formed in the bulk solution) and heterogeneous (nuclei are formed in the vicinity of surfaces) nucleation; for a wide range of species, comprising organic,^32^ inorganic^13^ and biological molecules,^33^ and for different volumes from microfluidic devices^34,35^ to industrial crystallizers.^32,36^ Nucleation is stochastic because the formation of new stable nuclei is an activated process, similar to a chemical reaction.^37^ The law of large numbers guarantees that a chemical reaction in a macroscopic system comprising a number of molecules of the order of the Avogadro number (6 × 10^23^ molecules, i.e., one mole) is a deterministic process. On the contrary, nucleation in a system of similar size leads to the formation of a number of particles many orders of magnitude smaller, hence the rare-event nature of the formation of a new nucleus cannot be ignored, and nucleation induces a potentially relevant variability in the properties of the crystallized suspension.^14^

Secondary nucleation, i.e., the formation of nuclei promoted by the presence of existing crystals, is also relevant.^38^ In agitated suspensions, the number of crystals formed by secondary nucleation is often several orders of magnitude larger than that of crystals formed by primary nucleation. In chiral systems, secondary nuclei are generally of the same handedness as their parent crystals. Therefore, the outcome of a crystallization process is governed by a relatively small number of stochastic primary nucleation events, sometimes just one per enantiomer in the case of chiral systems.^13,14,39,40^

Families of suspended crystals generated via primary or secondary nucleation from a supersaturated solution are populated by a series of stochastic nucleation events. Therefore, the properties of the process, e.g., the times of the first nucleation event and of the following ones and those of the crystal populations, e.g., the number of nuclei formed in a certain time interval, are stochastic variables, which depend on the prevailing nucleation rate and on the system volume.

The stochasticity of nucleation has two important consequences: (i) even under identical conditions, the nucleation of any substance occurs at different times and generates different numbers of crystals in different samples; (ii) in a racemic solution of a conglomerate-forming species, where both enantiomers are subject to exactly the same driving force, enantiopure crystals of the two enantiomers nucleate at different times forming different numbers of nuclei for each enantiomer.^12–14^ An important corollary of the latter point is that by the time the driving force is consumed and thermodynamic equilibrium is reached, the suspension will consist of two (possibly only slightly) different populations of enantiopure crystals; even if their masses happen to be identical, differences in the number of crystals and thus in mean crystal size are statistically inevitable. Upon spontaneous nucleation, symmetry-breaking has thus taken place.

### 2.3 Solid-State Deracemization Amplifies Any Initial Asymmetry

Solid-state deracemization consists in the amplification of an initial enantiomeric excess in suspensions of conglomerate-forming compounds in the presence of racemization in solution. This has been shown to happen either upon grinding at constant temperature or through temperature-cycling as well as in several other technical variations (see summary and references in the Introduction).

We have recently analyzed this process using a mechanistic model consisting of two symmetric ordinary differential equations, which describe the evolution of the suspension of the two populations of enantiopure crystals of a conglomerate-forming chiral species under the action of square-wave temperature cycles in the presence of a first-order interconversion reaction in solution.^29^ At high temperatures, crystals dissolve, while at low temperatures, crystals grow. With the model we have proven (and then confirmed experimentally) that an initially asymmetric suspension deracemizes whenever a simple condition is met, namely that crystal dissolution is faster than crystal growth.^29^ Whether this condition occurs or the opposite does is decided by the sign of a single dimensionless parameter (given by the difference between two parameters called *a*_d_ and *a*_g_, where the subscripts stand for dissolution and growth, respectively). We have proven that when such constraint is fulfilled, i.e., when (*a*_d_–*a*_g_) > 0, complete deracemization in favor of the initially major enantiomer occurs both in silico and in vitro under a broad range of conditions, namely: (i) however small the initial asymmetry, (ii) however small the amplitude of the temperature cycles, and (iii) whether temperature oscillations are either periodic or random.

Considerations related to the geometry of crystals, either growing or shrinking, demonstrate that crystal growth and final shape of the growing crystal are dominated by the slow-growing faces, while crystal dissolution and final shape of the dissolving crystal are dominated by the fast-dissolving faces.^41^ This conclusion has been reached theoretically^41–43^ and confirmed experimentally.^41^ Therefore, the condition that guarantees the occurrence of solid-state deracemization is not only simple, but indeed also ubiquitous.^29^

It is noteworthy that both Frank’s model and the solid-state deracemization model predict amplification of an initial asymmetry and complete asymmetry (homochirality) when a single parameter is positive (any initial asymmetry is symmetrized when the relevant parameter is negative in both models) (see the Supporting Information for more details).

Finally, it is also worth noting that the sign of *k*_3_ in the Frank model is, in principle, arbitrary since the model is an abstract representation of an idealized system, and one could easily envision situations where that sign is positive and situations where it is negative. In contrast, the sign of (*a*_d_–*a*_g_) in the solid-state deracemization model is essentially controlled by the sign of the difference between the rate constants of dissolution and growth. This difference must be positive because of the geometric arguments related to the shape evolution of crystals during growth and dissolution that we summarized above.

## 3 Results and Discussion

### 3.1 The Proposed Pathway to Homochirality

Based on the considerations in the previous section and building on the literature that we referenced there, we propose the following spontaneous pathway to homochirality in a prebiotic Earth, consisting of the stages evolving from an initial symmetric state, where a conglomerate-forming chiral species is in a perfectly racemic state, e.g., in solution in a pond, to a final fully homochiral state, where only enantiopure crystals of one enantiomer are present in the suspension:

1. Under natural circumstances, e.g., large temperature variations, the initial solution evolves toward either a supersaturated state (upon for instance solvent evaporation, the concentrations of both enantiomers increase beyond their solubility at the prevailing temperature) or an undercooled state (upon cooling, the temperature decreases below the equilibrium temperature corresponding to the prevailing enantiomer concentration).
2. Either solvent evaporation or cooling below the equilibrium temperature leads to supersaturation, which triggers stochastic primary nucleation of both enantiopure crystals, followed by crystal growth and secondary nucleation until the system evolves relatively quickly toward thermodynamic equilibrium at the prevailing conditions. Due to its stochasticity, such primary nucleation event breaks the initial symmetry and leads to the formation of two (possibly slightly) different populations of enantiopure crystals, suspended in a racemic solution.
3. Natural diurnal and seasonal temperature variations (or changes in system volume caused by evaporation and precipitations) set in motion the solid-state deracemization process described above, which in the presence of at least one species that catalyzes the (possibly very slow) racemization reaction, i.e., not enantioselective, amplifies the initial asymmetry and leads to a homochiral suspension. The handedness of such suspension is randomly selected by the stochastic primary nucleation event. This implies that on the prebiotic Earth, there might have been several homochiral pockets where enantiomers of opposite handedness prevailed.
4. Through interactions and mixing, as suggested by Frank, different pockets progressively homogenize, until “In the outcome, a single species should survive”^3^ and global homochirality should be achieved.

Though idealized, such a pathway through which homochirality might have evolved from a racemic prebiotic Earth is observable in the laboratory. As discussed in the next sections, we can confirm the plausibility of this pathway through both detailed simulations and laboratory experiments. It is worth noting that various biomolecules such as the amino acids threonine and asparagine crystallize as conglomerates^26^ and hence may undergo solid-state deracemization. Amino acids are of particular interest because they racemize in solution under a wide range of conditions, albeit slowly.^44,45^ In case racemization requires a catalyst to take place, there is no requirement regarding the chirality of the catalyst; the racemization of amino acids may be accelerated, for instance, both by (achiral) acidic conditions^44^ or by (chiral) enzymes.^22^ While we proved recently^29^ that the condition for deracemization is met no matter how slow the racemization reaction, we here choose a fast-racemizing species as model compound. This allows carrying out the large number of experiments required to quantitatively assess the effect of stochastic nucleation on symmetry-breaking with reasonable experimental effort.

### 3.2 Stochastic Simulations Predict Spontaneous Pathway to Homochirality

The evolution of a racemic solution of the enantiomers of a conglomerate-forming chiral species first toward a suspension containing ensembles of both enantiopure crystals, then ultimately toward a homochiral, i.e., enantiopure, suspension can be simulated using a stochastic population balance equation (PBE) model, that extends a methodology presented earlier.^46^ The model takes into account that crystals are discrete entities, i.e., only an integer number of crystals may exist, and that nucleation is stochastic. The phenomena that are considered are the primary and secondary nucleation of crystals, which are described as stepwise Poisson processes, the growth and dissolution of crystals, and the chemical interconversion reaction between the two enantiomers in solution. The model equations, their numerical implementation, and the choice of model parameters are discussed in detail in Section 5.2.

The model is used to perform simulations, each comprising two distinct phases. During the first phase, a racemic solution evolves into a suspension triggered by linear cooling. Cooling lowers the solubility of both enantiomers and generates the driving force for primary nucleation and subsequent growth and secondary nucleation of crystals. During the second phase, the suspension is subjected to square temperature cycles, which amplify the chiral asymmetry of the suspension observed at the end of the first phase and drive it to homochirality.

An example of such a simulation is illustrated in Figure 1, where the top (whole simulation) and the right (first temperature cycle) panels show the temporal evolution of solubility (black line, initially decreasing due to linear cooling of the solution and then fluctuating due to square-wave temperature cycles) and solute concentrations in solution (blue and red lines). The bottom panel shows the evolution of the enantiomeric excess for ten independent simulations carried out with the same operating parameters and initial conditions, whereby the black line corresponds to the reference simulation illustrated in the other two panels, and the blue and red markers indicate the times of the first nucleation events for the two enantiomers. The enantiomeric excess, *ee*, is defined as the ratio between the difference and the sum of the masses of the suspended crystals of the two populations, i.e., *ee* = (*n*_1_–*n*_2_)/(*n*_1_ + *n*_2_); *ee* = 0 corresponds to a racemic system, where *n*_1_ = *n*_2_; *ee* = −1 and *ee* = 1 correspond to enantiomeric purity in enantiomer 2 and in enantiomer 1, respectively.

First, let us consider the reference simulation. After 1 h of cooling to reach the specified lower temperature level (marked with a vertical dashed line in all diagrams), the suspension exhibits an enantiomeric excess that favors in this case enantiomer 2, i.e., *ee* < 0. This outcome has resulted (i) from an early nucleation of crystals of enantiomer 2, (ii) followed by growth and conversion of enantiomer 1 into enantiomer 2, whose concentration has been depleted by crystal formation and growth, and (iii) by nucleation and consequent growth of enantiomer 1 as well. The shoulder in the evolution of *c*_1_ results from two distinct mechanisms: initially, the concentration decreases due to conversion into enantiomer 2 (which had nucleated first); as soon as some crystals of enantiomer 1 have formed, its own crystallization depletes the concentration in solution to the solubility value. Note that the definition of enantiomeric excess is only meaningful when crystals are present; hence, the *ee* line in the lower plot starts at the time when the first primary nucleus forms. Given that this first nucleus is of a certain handedness (that of enantiomer 2 in this case), the suspension is enantiopure at this point in time, and it remains so, until a primary nucleus of the other enantiomer is formed. The asymmetry at the end of cooling is amplified by temperature-cycling in an oscillatory manner until homochirality is achieved. With reference to the right panel, it is worth noting that during dissolution at high temperature (high solubility), the majority enantiomer 2 has a higher concentration and is converted into enantiomer 1, whereas the opposite occurs during growth at low temperature (low solubility). As demonstrated elsewhere, the areas between the red and the blue lines indicate the extent of conversion: as the area between the two lines during growth is larger than that during dissolution, the net conversion during one cycle favors the majority enantiomer 2.^29^

Then, let us consider all of the ten simulations, whose enantiomeric excess is plotted in the lower panel of Figure 1; the same *ee* profiles are plotted in different colors over a longer time in the top left panel of Figure 2. A few observations are worth making. First, with reference to the latter figure, all ten simulations attain homochirality, mostly after the system experiences temperature-cycling. Second, the handedness of the homochiral state and the time needed to attain deracemization differ in the ten simulations, namely between ca. half an hour and ca. 4 h. Third, such differences are a consequence of the stochastic components of the system, which are incorporated in the model and manifest themselves most clearly during the first cooling phase of the process, when nucleation occurs (see Figure 1). Fourth, all simulations behave qualitatively like the reference one, with either enantiomer 1 or enantiomer 2 nucleating first, thus generating a pure suspension, i.e., *ee* = ±1; as soon as the other enantiomer also nucleates the enantiomeric excess evolves toward zero; the *ee* value at the end of cooling and at the onset of the temperature cycles results being very different in these ten simulations carried out at exactly the same conditions. Finally, it is apparent that the deracemization time is larger when the absolute *ee* value is smaller at the onset of temperature cycles.

**Figure 1:**
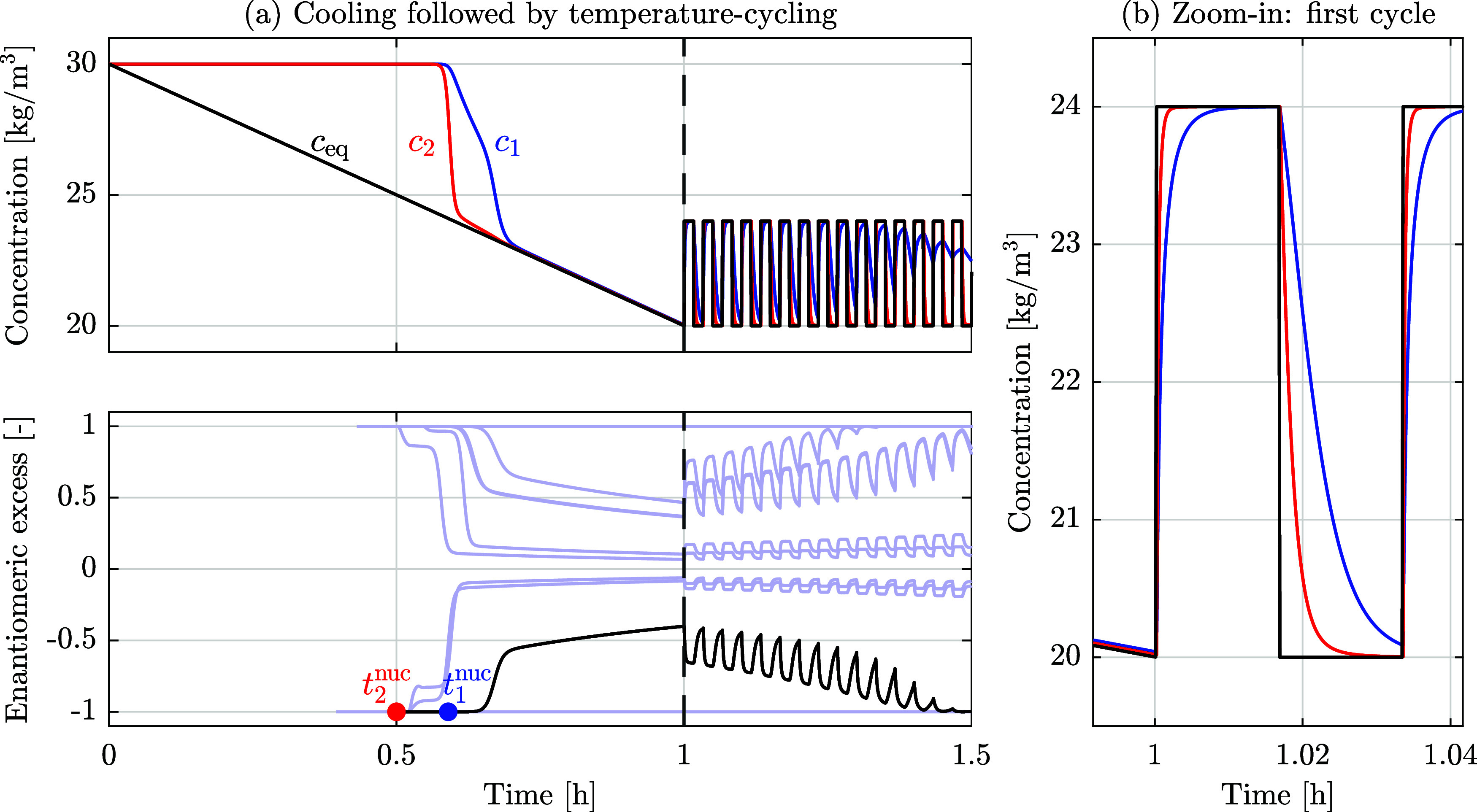
Stochastic simulations starting from racemic solution with a decrease in solubility (as induced by cooling) from 30 kg/m^3^ to 20 kg/m^3^ in 1 h. (a) Top: evolution of solubility (black) and enantiomer concentrations (blue and red) in solution during a single simulation. The concentrations in solution decrease as crystals are formed and material interconverts between the two enantiomers. Bottom: Evolution of enantiomeric excess for ten independent simulations, whereby the black line corresponds to the simulation shown in the top panel. (b) Zoom-in focused on the first temperature cycle. The concentration of enantiomer 2 (red) in solution changes faster than that of enantiomer 1 (blue), because more crystals of enantiomer 2 have formed during the initial cooling phase (negative enantiomeric excess). This difference is a prerequisite of deracemization.

**Figure 2:**
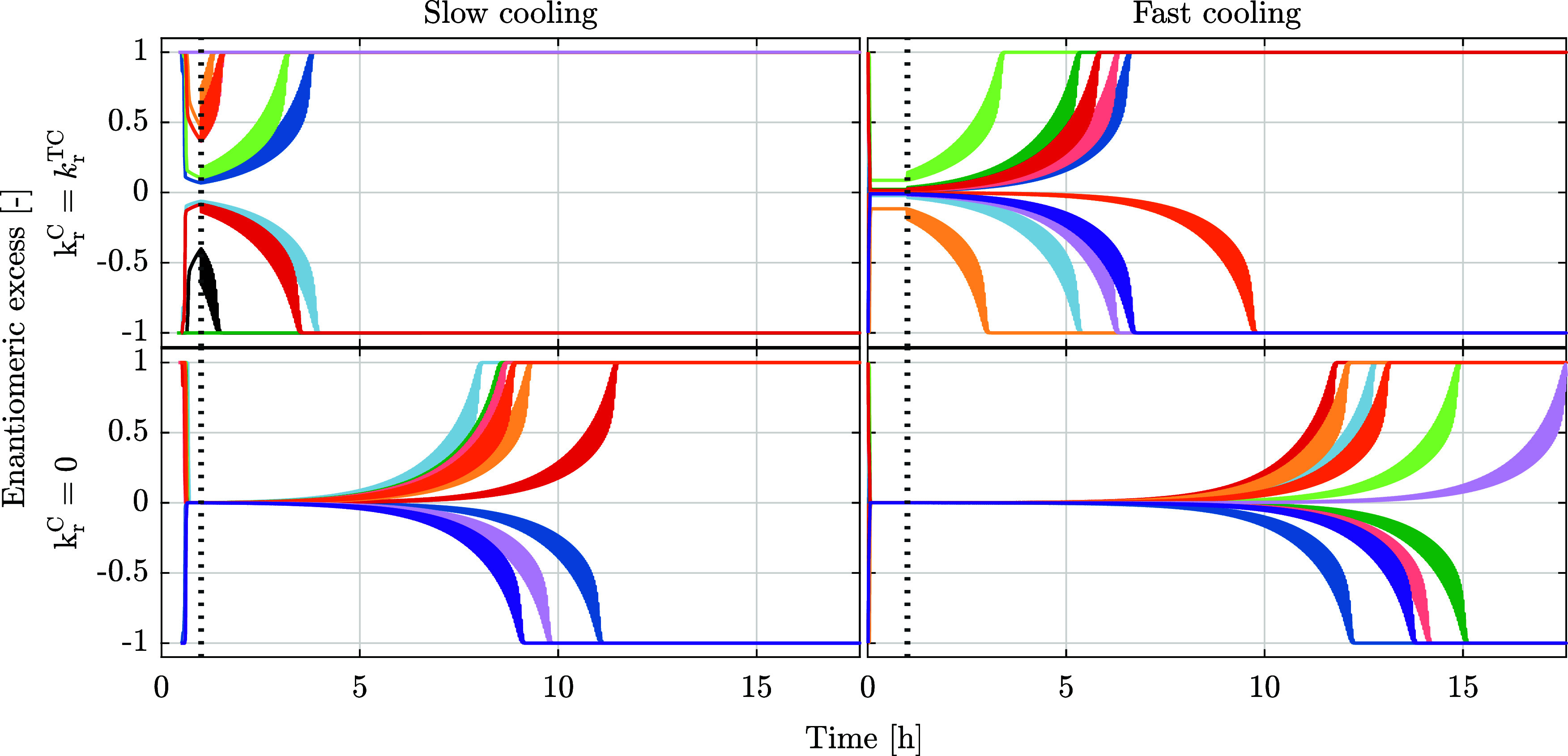
Stochastic simulations starting from racemic solution with a decrease in solubility (as induced by cooling) from 30 kg/m^3^ to 20 kg/m^3^ in 1 min (right) or in 1 h (left). The chemical reaction has either been turned on during the entire simulations (top) or turned on during temperature-cycling only (bottom), i.e., turned off during the initial cooling phase. The vertical dashed line indicates the onset of temperature-cycling, which takes place after 1 h. The lines correspond to ten independent simulations per panel. The simulations shown in the top left panel are identical to those shown in Figure 1.

In some cases (the pink and green curves in Figure 2, top left panel), the suspension remains enantiopure throughout the cooling phase; after the first primary nucleation event, growth, and subsequent secondary nucleation deplete the concentration of the nucleated enantiomer in the solution. This leads to a concentration difference between the two enantiomers (as seen in Figure 1, top left panel), which in turn drives the conversion reaction toward this same enantiomer and hence reduces the supersaturation of the other enantiomer that has not nucleated yet and even prevents its primary nucleation.

It is now worth looking at three other sets of simulations, carried out by varying the cooling rate and by switching the racemization reaction on and off during cooling. In the upper right panel of Figure 2, the cooling rate is 60 times faster than in the top left panel of the same figure. It is apparent that the enantiomeric excess at the end of cooling in the case of fast cooling is much smaller than in the case of slow cooling, and that the times to deracemization are accordingly longer, namely, between ca. 3 and almost 10 h. In the two lower panels of Figure 2, the racemization reaction is turned off during cooling; this corresponds to an experimental (or natural) scenario in which the catalyst of the racemization reaction is added at the beginning of the temperature cycles, while it is not present during crystallization. As a consequence, the times to deracemization are significantly larger, in both cases of slow (from approximately 0.5 to 4 h, to approximately 8 to 12 h) and fast cooling (from approximately 3 to 10 h, to approximately 12 to 20 h). In both cases, the absence of the conversion reaction during cooling leads to very small asymmetry levels at the end of cooling and at the onset of temperature cycles; in fact, the enantiomeric excess is zero, and the asymmetry is limited to tiny differences in specific features of the particle size distributions of the two enantiomers.

These results can be rationalized by considering that during cooling, the supersaturation of each enantiomer in solution is affected by three mechanisms that drive its change. Cooling increases supersaturation; the faster the cooling, the higher the supersaturation. Crystal nucleation and growth deplete supersaturation of the crystallizing enantiomer. The interconversion reaction depletes the supersaturation of the enantiomer that has crystallized less (the minority enantiomer in the solid phase) to the advantage of the enantiomer that has crystallized more (the majority enantiomer). It follows (i) that faster interconversion during cooling enhances asymmetry and vice versa and (ii) that faster cooling reduces asymmetry at the end of cooling, hence leading to longer deracemization times and vice versa. The quantitative results illustrated in Figure 2 are perfectly consistent with these qualitative considerations.

For stochastic simulations, it is possible not only to monitor the enantiomeric excess, as shown in Figure 2 and done in experiments as well, but also to track the evolution of the full particle size distributions, including the number of primary nuclei that form. This provides all of the information required to study in detail the asymmetry that enables deracemization and leads to homochirality. For the four sets of operating conditions considered in Figure 2 and based on 500 independent simulations for each set, Figure 3 shows the cumulative distribution of three quantities: (a) the number of primary nuclei of enantiomer 1 at the end of the initial cooling; (b) the ratio of the overall surface areas of the two family of enantiopure crystals, ξ, defined as the surface of enantiomer 2 over the surface of enantiomer 1, at the end of cooling; (c) the time to achieve homochirality, defined starting from the onset of the cooling phase.

**Figure 3:**
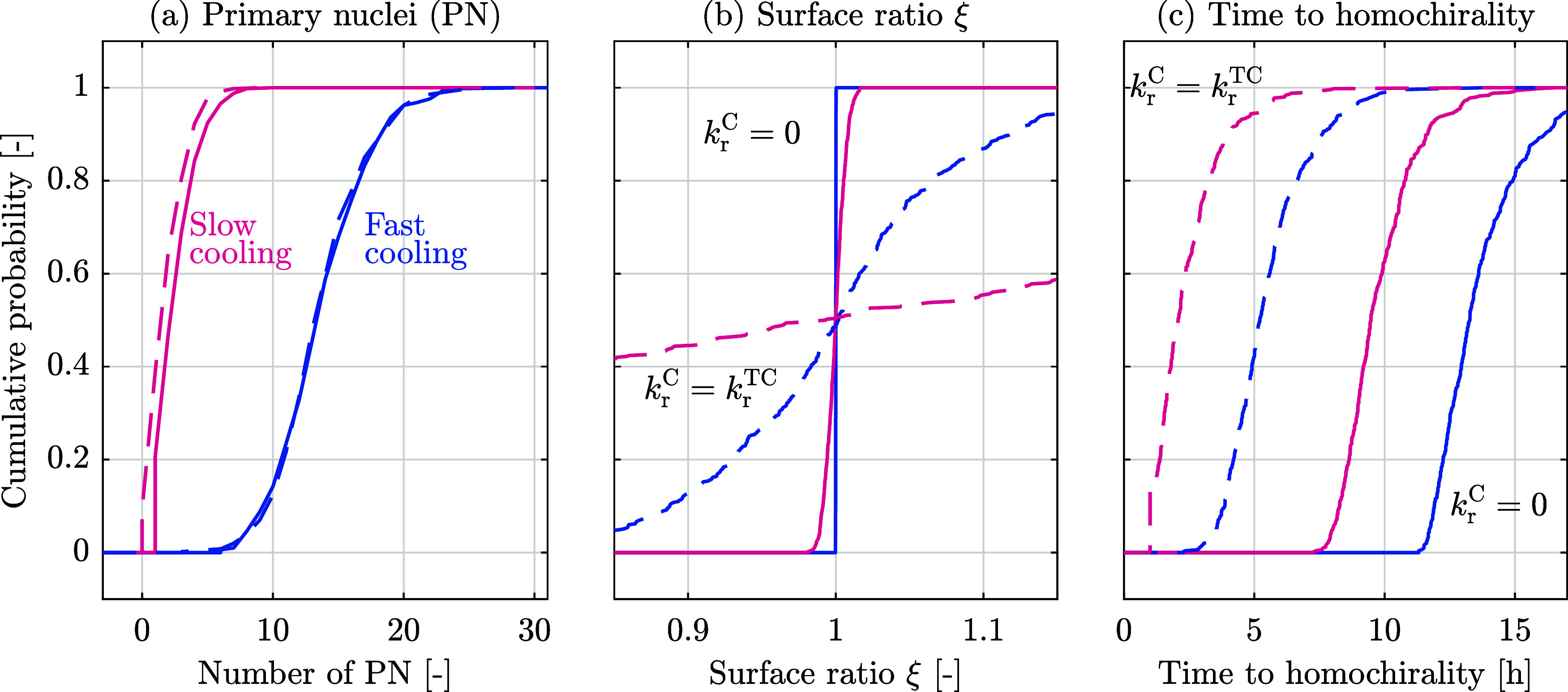
Cumulative distributions (a) of the number of primary nuclei of enantiomer 1, (b) of the surface ratio ξ, and (c) of the time required to reach homochirality. All distributions were constructed using data obtained from 500 independent simulations. The four lines correspond to the four conditions shown previously: slow and fast cooling (colors) and presence (dashed lines) or absence (solid lines) of racemization reaction during the initial cooling phase.

Figure 3a shows that the number of primary nuclei is insensitive to the presence or absence of the catalyst during cooling, while many more nuclei are formed when cooling is faster. It is worth noting that in the case of slow cooling with chemical reaction, there are indeed simulations in which only a single primary nucleus forms. Figure 3b shows that in the absence of the interconversion reaction during cooling, the asymmetry is very small, that is, ξ ≈ 1 and exhibits a very small variability between simulations; this is due to the fact that without racemization the two enantiomers crystallize independently of one another. The surface ratio exhibits a large variability instead when the interconversion reaction is present during cooling and may exert its role in enhancing the asymmetry between the two populations of crystals at the end of cooling. Figure 3c shows that the time to achieve homochirality is only a factor larger when there is no catalyst during cooling than where there is catalyst: the average time is about 4 times larger and about 2.5 times larger in the cases of slow and fast cooling, respectively. This follows from the exponential nature of the chiral amplification during solid-state deracemization: we derive an analytical solution for the factor at which a temperature cycle amplifies the asymmetry in Section 5.1, which is exact in the limit of small asymmetries; for the conditions used in the simulations, the asymmetry is predicted to increase by a factor of 2 after every 25 cycles, i.e., every 50 min. This explains why, independent of the initial asymmetry, all suspensions deracemize within a certain factor of time. Naturally, this exponential nature is of immediate relevance to the emergence of homochirality on the prebiotic Earth, as it provides evidence that solid-state deracemization may proceed in reasonable time scales no matter how small the initial asymmetry generated by stochastic nucleation is.

### 3.3 Experiments Confirm the Spontaneous Emergence of Homochirality

Next, we experimentally confirm that the interplay of stochastic nucleation and solid-state deracemization enables the spontaneous emergence of homochiral suspensions from racemic solutions, as predicted by the stochastic simulations shown above. We carried out experiments with the conglomerate-forming species *N*-(2-methylbenzylidene)-phenylglycine amide **(NMPA)**, an imine derivative of phenylglycine, in the presence of the base 1,8-diazabicyclo[5.4.0]­undec-7-en **(DBU)** that catalyzes its racemization in solution,^18,47^ at mL scale (see the detailed experimental methods in Section 5.3). In line with the simulations, experiments comprise first a cooling phase in which a clear racemic solution crystallizes and second a temperature-cycling phase, in which the enantiomeric excess is amplified until homochirality is achieved. In each experiment, the evolution of the enantiomeric excess was monitored in eight independent crystallizers.

We investigated the effect of three operating variables during the initial cooling phase, namely, (i) the cooling rate (two levels), (ii) the catalyst concentration (two levels), and (iii) the initial concentration (three levels). The operating conditions are reported in Table 1, while the corresponding time evolution of the enantiomeric excess starting from the beginning of the temperature cycles to the achievement of homochirality is shown in Figures 4 and 6. The enantiomers of NMPA are denoted as “L” and “D”, so that the enantiomeric excess is defined as *ee* = (*n*_L_ – *n*_D_)/(*n*_L_ + *n*_D_) (note that in the simulations, the enantiomers were labeled with “1” and “2”).

**Table 1:**
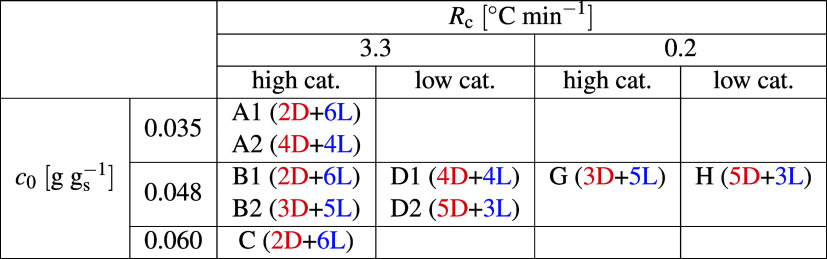
Experimental Conditions Explored in This Work^a^

a The numbers in parentheses indicate the final handedness (D or L) achieved in the eight crystallizers operated in each experiment.

**Figure 4:**
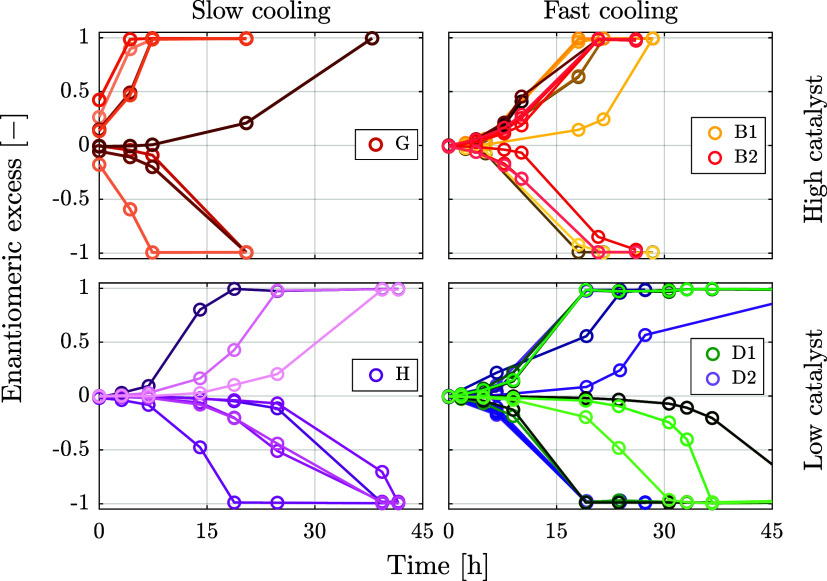
Effect of the cooling rate and the catalyst concentration on the enantiomeric excess of crystalline suspensions formed upon cooling. Each panel shows the evolution of enantiomeric excess of eight or sixteen crystallizers under identical experimental conditions. Both increasing the cooling rate and decreasing the catalyst concentration decrease the chiral asymmetry that forms upon the initial cooling phase and hence slow down deracemization.

Figure 4 illustrates the effect of the cooling rate and of the catalyst concentration at constant initial concentration, *c*_0_ = 0.048 *gg*_s_^–1^, and mirrors the simulations shown in Figure 2. The top and bottom parts are at high and low catalyst concentrations, respectively, whereas the left and right parts are at slow and fast cooling, respectively. For fast cooling conditions, two experiments each were carried out; therefore, in these cases, there are two sets of eight *ee* plots, instead of only one set.

The first observation is that all crystallizers in all nine experiments, i.e., 72 individual crystallization processes, have reached homochirality. Although obviously eight crystallization processes are not enough to build a statistically significant set of data, it is apparent that the number of experiments achieving D or L handedness is random (from 2 and 6 L in one set of eight experiments to 5 and 3 L in another). In fact, the probability of observing a distribution as extreme as ours (30 D and 42 L) lies at 19%, assuming a binomial distribution with 72 trials and 50% probability of success. We interpret this observation as a confirmation that the crystallization process and the achievement of homochirality take place as conjectured by the theory and confirmed by the stochastic simulations, i.e., through spontaneous symmetry-breaking triggered by stochastic nucleation, followed by amplification through solid-state deracemization via temperature cycles.

The second observation is that, when under the same conditions two sets of experiments are repeated, the results are similar from a semiquantitative point of view, as observed in the case of the yellow and red sets in experiments B1 and B2, and in the case of the green and purple sets in experiments D1 and D2. More cannot be said again because of the lack of statistical significance of the set of eight experiments per set obtained here.

The third observation is that the trends observed experimentally are the same as those observed in the simulations reported in Figure 2: increasing the cooling rate at the same catalyst concentration or decreasing the catalyst concentration during cooling at the same cooling rate leads to overall longer deracemization times.

The final observation is that after cooling and before temperature cycles, only experiment G exhibits measurable values of the enantiomeric excess, i.e., up to almost 0.5, whereas in the other three cases, the enantiomeric excess is smaller than 0.01, i.e., below the detection limit of the method. This result is also consistent with what is observed in Figure 2 and highlights how even the smallest asymmetry that stems from stochastic nucleation during the cooling phase of the process is amplified by temperature cycles to ultimate homochirality.

### 3.4 From Local to Global Homochirality

Through the combination of simulations and experiments, we have established that homochirality spontaneously emerges at a local level in crystalline suspensions. Here, we show that such *local* homochirality serves as a starting point for the emergence of homochirality on a *global* level. To this end, we study first the effect of volume (through simulations) and second the effect of the initial concentration (through both simulations and experiments) on deracemization. It is worth noting that we do not know the amount of solute that was present when homochirality first emerged on Earth; hence, by demonstrating that local homochirality emerges independently of how much solute is present, we provide evidence that this mechanism is indeed of prebiotic relevance. Third and finally, through experiments, we investigate the effect of merging suspensions that have reached local homochirality, a phenomenon that plausibly has taken place on the prebiotic Earth.

The simulations shown in Figure 5 demonstrate the effects of volume and solution concentration on the deracemization process, with ten independent simulations shown in each panel. The reference case (b, d) corresponds to a volume of *V* = 50 mL and a concentration of *c*_0_ = 30 kg/m^3^. The top panels correspond to three volumes at the reference concentration. As can be seen, decreasing the volume (*V* = 1 mL) leads to a larger initial asymmetry and faster deracemization; in fact, eight out of ten simulations achieved homochirality already during the cooling phase. This is because in most simulations, only a single primary nucleus forms due to the linear relation between the nucleation frequency (that is, the expected number of nuclei that form per unit time) and the volume, as to *K* = *JV*. In contrast, increasing the volume (*V* = 5000 mL) leads to smaller initial asymmetries (due to more primary nucleation events) and hence longer deracemization times.

**Figure 5:**
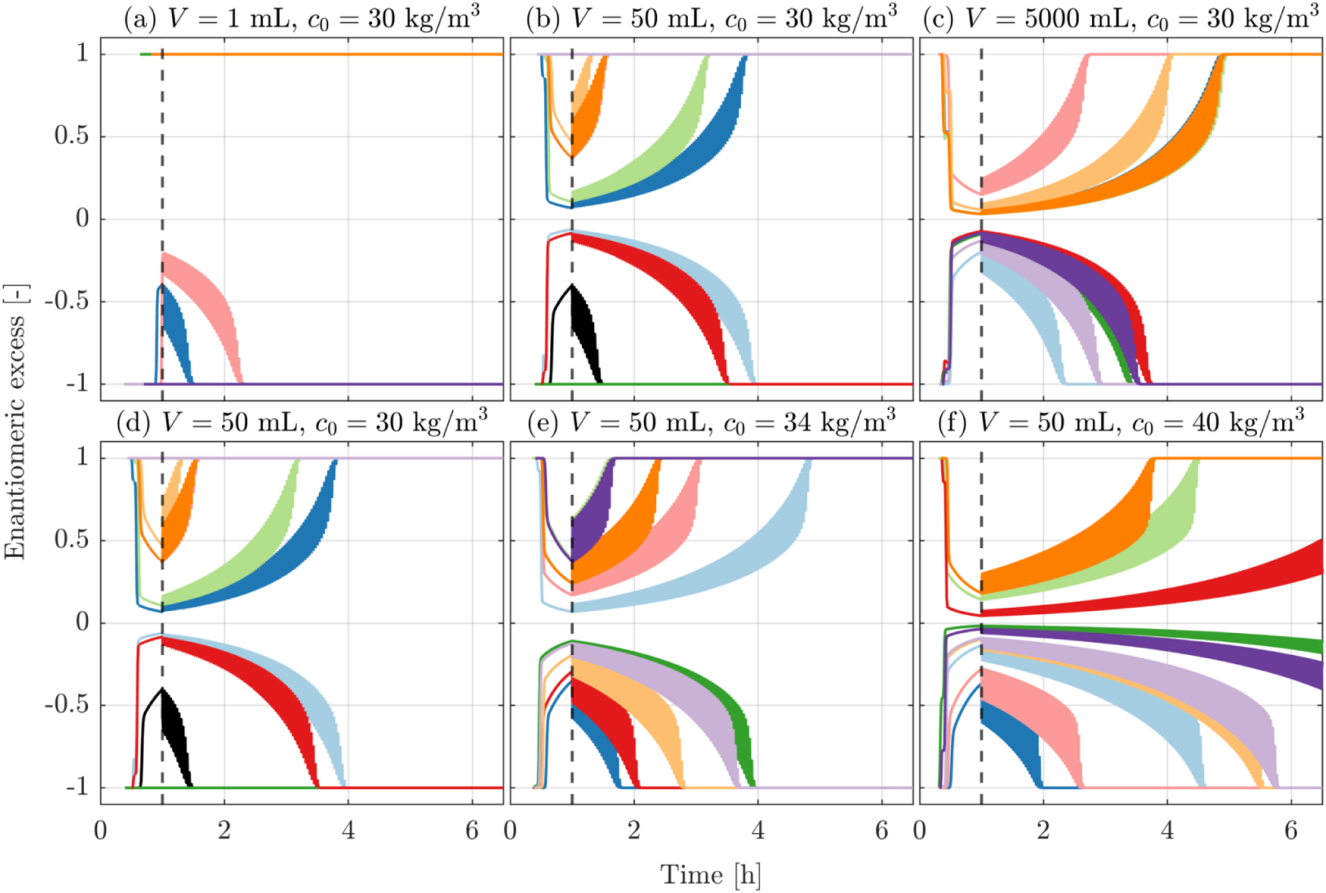
Top: Effect of volume on deracemization, as shown in stochastic simulations starting from racemic solution with a decrease in solubility from 30 to 20 kg/m^3^ in one hour. Three volumes are considered: 1 mL (a), 50 mL (b), and 5000 mL (c). Bottom: Effect of initial concentration on deracemization, as shown in stochastic simulations for a volume of 50 mL starting from racemic solution with a decrease in solubility from (d) 30 kg/m^3^, (e) 34 kg/m^3^, and (f) 40 to 20 kg/m^3^ in one hour. Ten independent simulations are shown in each panel, whereby the simulations shown in panels (b) and (d) are identical.

Next, we consider the effect of solution concentration (bottom part of Figure 5). Simulation parameters for temperature-cycling were chosen such that for all initial concentrations, the same fraction of crystals dissolves upon raising the solubility (i.e., 40%). For instance, in the reference case, the initial concentration is *c*_0_ = 30 kg/m^3^ and temperature-cycling proceeds between solubility values of 20 and 24 kg/m^3^. Increasing the initial concentration to 34 and 40 kg/m^3^ leads to smaller initial asymmetries and longer deracemization times. This is further supported by the observation that no simulations achieved homochirality during cooling at the higher concentrations. There are multiple reasons for this behavior, as summarized in the following.

First, higher initial concentrations allow for higher supersaturations to be achieved during the cooling phase, which, in turn, may result in a larger number of primary nuclei formed (as the nucleation rate strongly increases with supersaturation). As discussed in Section 3.2, a larger number of primary nuclei is connected to a smaller initial asymmetry and hence slower deracemization. Second, for a given number of primary nuclei, a higher initial concentration implies that more material is available for further secondary nucleation and crystal growth; this again leads to a smaller initial asymmetry. Third, the dynamics of deracemization during temperature-cycling itself depends on the amount of crystals present in the suspension: the concentrations in solution react more quickly to temperature/solubility changes, the more dense the suspension is. We refer to our earlier work^29^ for a detailed discussion on the effects that govern the deracemization rate.

It is worth noting that the trends predicted in the simulations for the effect of the initial concentration are confirmed by experiments, as shown in the top part of Figure 6. The three panels correspond to experiments at different initial concentrations (for the sake of comparison, experiment B1 is shown in this figure again). As expected, increasing the solute concentration decreases the asymmetry after cooling and slows down deracemization. In experiments conducted at the lowest concentration, the average absolute value of enantiomeric excesses at the end of the cooling phase of eight crystallizers ( $\overset{®}{{\mathrm{ee}}_{0}}$ ) are 0.20 for A1 and 0.15 for A2. For the other experiments, $\overset{®}{{\mathrm{ee}}_{0}}$ is smaller than the limit of quantification, which is on the order of 0.01. Finally, the lower part of Figure 6 reports mixing experiments, which are aimed at demonstrating that the evolution from local homochirality to a global one occurs spontaneously. For the sake of brevity, only one case is presented, namely, that of experiment B1; the same protocol applied to other experiments yielded conceptually identical results. In this case, starting from a racemic solution (represented by the gray circles in the left side of the bottom left panel), the experiment yielded 2 suspensions with D handedness and 6 with L handedness. Then, the knockout protocol is applied for a first time (step a) to pairs of suspension chosen randomly, which yields 1 suspension and 3 suspensions with D and L handedness, respectively, and then for a second time (step b), which yields one suspension for each handedness. When these two are mixed and temperature cycles are applied (step c), *global* homochirality with L handedness is achieved. The panel on the right side of the lower part of the figure shows the evolution of the enantiomeric excess for the four cases where two suspensions with opposite handedness have been mixed.

**Figure 6:**
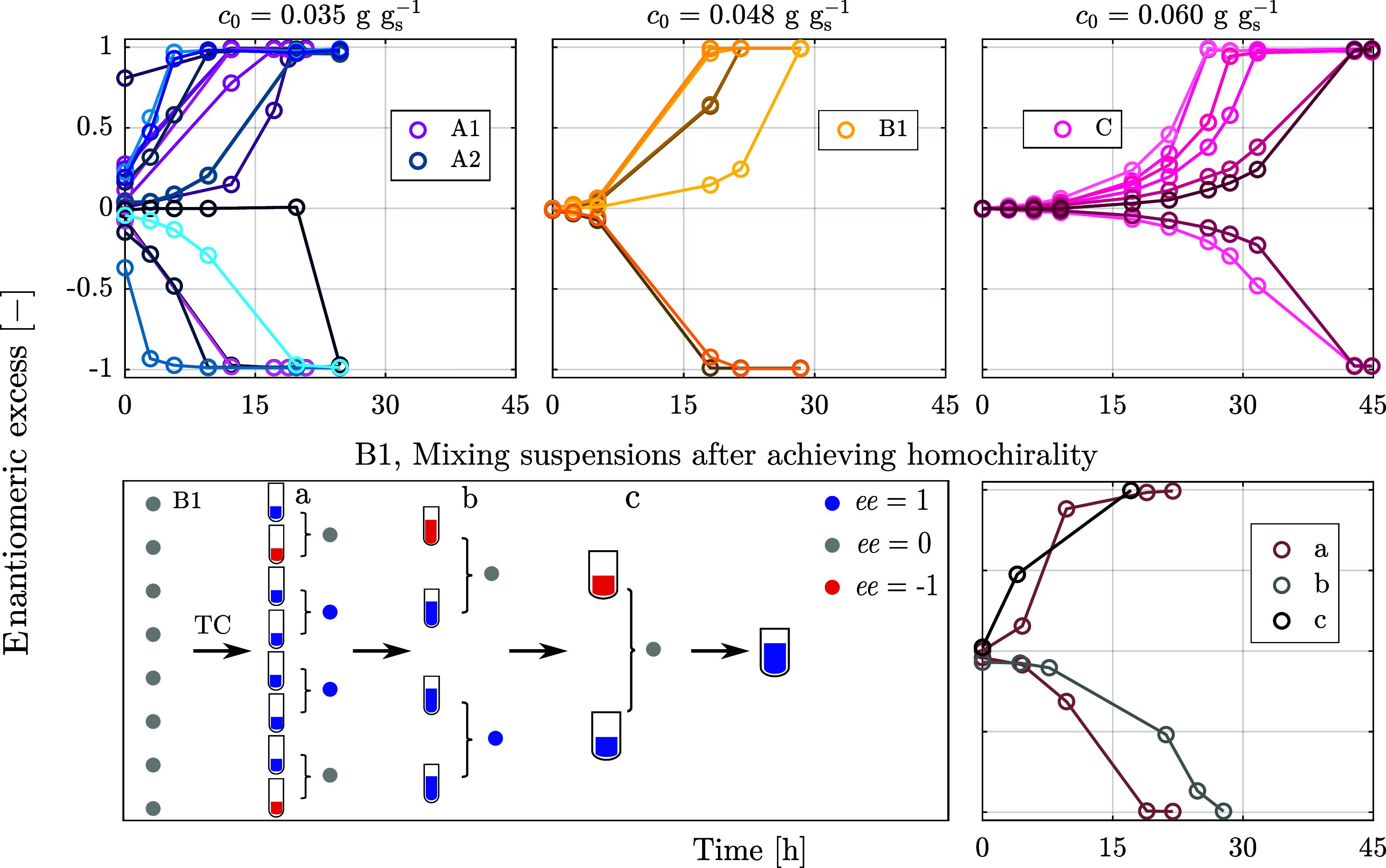
Effect of the initial concentration (top panels): Higher initial concentration results in smaller enantiomeric excess of crystals formed upon cooling, thus longer deracemization time thereafter. The bottom panels report the mixing study that was performed for the eight crystallizers of experiment B1. The circles to the left of the crystallizers as well as the filling color inside the crystallizers, refer to chirality: gray indicates that no handedness prevails, blue that the system is enantiopure in L crystals, and red in D crystals. The random mixing of enantiopure suspensions under the presence of racemization reaction and temperature fluctuations ultimately results in single-handedness of the final suspension.

This mixing and deracemization protocol implements the conceptual path conjectured by Frank from many local homochiral states of different handedness, to an ultimate single global handedness.^3^

## 4 Conclusions

Here, we have developed a stochastic model and designed experiments to demonstrate a plausible pathway toward homochirality that a conglomerate-forming chiral species can experience in the laboratory today and might have experienced on Earth before life emerged. Such a pathway starts from a racemic solution of the two enantiomers and involves two steps, namely: (i) spontaneous symmetry-breaking caused by stochastic nucleation driven by supersaturation, which can be caused by quite common climatic events; (ii) asymmetry amplification caused by temperature fluctuations experienced by the suspension of the two families of enantiopure crystals (because of diurnal, seasonal, or geological temperature variations), in the presence of an agent that enables the interconversion (racemization) reaction (this is called solid-state deracemization). As such a process leads to homochiral suspensions with different local handedness, we have also demonstrated a third step that leads to global homochirality, in the laboratory and in principle also on the prebiotic Earth, through a knockout process driven by casual encounters between families of crystals with opposite handedness under conditions where solid-state deracemization may take place. We have shown that stochastic simulations and laboratory experiments, using the chiral compound *N*-(2-methylbenzylidene)-phenylglycine amide (NMPA), exhibit the same features and the same trends when changing the operating conditions during the initial symmetry-breaking phase driven by linear cooling of the solution, either by enhancing supersaturation (by faster cooling or higher solute concentration) or by slowing down the racemization reaction. These observations, together with their rationalization based on theoretical considerations, provide further confirmation of the physical and chemical viability of the proposed mechanism.

It is worth noting that the plausibility of the mechanism proposed for the establishment of homochirality is supported by the fact that both stochastic nucleation and solid-state deracemization by temperature cycles are well established. They are combined in the context of the emergence of homochirality for the first time here. This study represents a convincing embodiment of the pioneering theory and conjectures proposed by Frank in 1953, based on simple and ubiquitous phenomena involving suspensions of conglomerate-forming chiral compounds.

## 5 Materials and Methods

### 5.1 Theoretical Analysis of Solid-State Deracemization

In this section, we prove the exponential nature of the chiral amplification through temperature-cycling in the limit of small chiral asymmetries using a mechanistic solid-state deracemization model. The model describes how the suspension of the two populations of enantiopure crystals of a conglomerate-forming chiral species evolve under the forcing action of square-wave temperature cycles (that trigger crystal growth and dissolution at low and high temperatures, respectively) in the presence of an interconversion reaction in solution (racemization reaction).^29^ In a well-stirred batch crystallizer (*i* = 1, 2, *j* = 3 – *i*), the material balances are

1 $$\frac{dc_{i}}{dt}+\frac{dn_{i}}{dt}=-k_{r}\left(c_{i}-c_{j}\right)$$

where c_*i*_ and *n*_*i*_ = *m*_3,*i*_ *k*_v_ρ_c_ denote the mass of solute in the liquid and in the solid phase, both defined either per unit mass of solvent or per volume. *k*_r_(*T*) is the temperature-dependent rate constant of racemization, which is assumed to be a symmetric first-order reversible reaction. The variable *m*_3,*i*_ is the third moment of the particle size distribution of enantiomer *i*, *k*_v_ is the volume shape factor, and ρ_c_ is the crystal density. The *k*-th moment of the particle size distribution is defined as

2 $$m_{k,i}=\int_{0}^{\infty }L^{k}f_{i}\left(L\right)dL$$

and through this definition, eq 1 is coupled to the population balance equations of the two populations of crystals.

As the Frank model, also this model reduces to the following two ordinary differential equations, when assuming that crystals grow and dissolve at constant surface area, i.e., constant value of the second order moments *m*_2,*i*_, with *m*_2,1_ > *m*_2,2_, and that their rates of growth and dissolution are linear in the supersaturation (all these assumptions can be relaxed without changing the essence of the results that are important here^29^):

3 $$\frac{dc_{i}}{dt}+3\rho_{c}k_{v}m_{2,i}k_{m}\left(c_{i}-c_{\mathrm{eq}}\right)=-k_{r}\left(c_{i}-c_{j}\right)$$

Here, the parameters *k*_m_(*T*) and *c*_eq_(*T*) are the temperature-dependent rate constant of growth or dissolution and the solubility, respectively. The whole process is simulated by cycling the system of equations through the temperature square waves, which results in the enantiomer concentrations in solution attaining a cyclic steady state and in the masses of the two populations of crystals undergoing changes due to the growth and dissolution of crystals, as well to the racemization reaction, which converts the majority enantiomer in solution into the minority one.

For the sake of brevity, we do not repeat all the details about the solid-state deracemization model, as the interested reader can find them in our recent paper.^29^ Nevertheless, it is worth reporting the key argument for the purpose of this theoretical analysis.

The change in mass of the major enantiomer crystals (index 1, target enantiomer) per unit mass of solvent throughout a single cycle, Δ*n*_cyc_, is defined as the integral over the growth step (at *T*_g_) and the dissolution step (at *T*_d_, with *T*_g_ < *T*_d_) of the rate of the conversion reaction (*k*_r_) from the minor enantiomer (index 2, undesired enantiomer) to the major enantiomer (see eq 4 of ref 29):

4 $$\Delta n_{\mathrm{cyc}}=\int_{g}k_{r}\left(T_{g}\right)\left(c_{2}-c_{1}\right)dt+\int_{d}k_{r}\left(T_{d}\right)\left(c_{2}-c_{1}\right)dt$$

Note that growth favors the major enantiomer, whereas dissolution favors its antimer; hence, the first integral is positive and the second is negative. We have recently derived an analytical solution for Δ*n*_cyc_ (see ref 29 for details):

5 $$\Delta n_{\mathrm{cyc}}=\frac{\xi a_{d}a_{g}\left(a_{d}-a_{g}\right)\left(\Delta x_{2}-\xi \Delta x_{1}\right)}{\det\left({\underline{\mathrm{A̲}}}_{d}\right)\det\left({\underline{\mathrm{A̲}}}_{g}\right)}$$

We note that the corresponding eq 6 in ref 29 contains a typo, i.e., the prefactor *a*_d_ *a*_g_ was omitted in the print; however, all calculations in ref 29 were carried out using the correct equation. In eq 5, ξ is defined as the ratio of the surface areas available for growth and dissolution between the minority family of enantiopure crystals and the majority one, hence ξ = *m*_2,2_/*m*_2,1_ < 1; the proportionality constant is always positive and the parameter *a*_m_ is proportional to the ratio between the rate constant of growth or dissolution and that of racemization at the relevant temperature, i.e., *a*_m_ ∝ *k*_m_(*T*_m_)/*k*_r_(*T*_m_), with m = g for growth, or m = d for dissolution. The matrices are defined as

6 $${\underline{\mathrm{A̲}}}_{m}=\left[\begin{array}{ll} -\left(a_{m}+1\right) & 1\\ 1 & -\left(a_{m}\xi +1\right) \end{array}\right]$$

Δ*x*_*i*_ denotes how much the concentrations in solution change for the two enantiomers during a cycle, whereby Δ*x*_1_ = Δ*x*_2_ = Δ*c*_∞_ in the case that the times at high and low temperatures are sufficiently long that the concentrations in solution approach their equilibrium value (i.e., the solubility) during the cycle. It is worth noting that both simulations and experiments (consider temperature-cycling experiments carried out previously^48^ under similar operating conditions where we observed that increasing the cycle time did not affect the cycle efficiency) that have been carried out in this work correspond to this case. This case is of particular interest because the expression for Δ*n*_cyc_ simplifies, which allows the derivation of a chiral amplification factor. Δ*n*_cyc_ becomes

7 $$\Delta n_{\mathrm{cyc}}=\frac{\xi \left(1-\xi \right)\left(a_{d}-a_{g}\right)a_{d}a_{g}\Delta c_{\infty }}{\det\left({\underline{\mathrm{A̲}}}_{d}\right)\det\left({\underline{\mathrm{A̲}}}_{g}\right)}=\phi K\left(a_{d},a_{g},\phi \right)$$

where in the second equality, we introduce the parameter ϕ = 1 – ξ, which characterizes the extent of the chiral asymmetry, i.e., it is zero for a perfectly symmetric suspension and increases with increasing asymmetry, and the parameter *K*(*a*_d_, *a*_g_, ϕ) that contains all other functional dependencies. Eq 7 serves as a starting point for the derivation of the change in the chiral asymmetry for a single cycle. First, the change in enantiomeric excess between cycles *k* and *k* + 1 is

8 $$\Delta \mathrm{ee}=ee_{k+1}-ee_{k}=\frac{2\Delta n_{\mathrm{cyc}}}{n_{\mathrm{tot}}}$$

using the definitions *ee* = (*n*_1_–*n*_2_)/(*n*_1_ + *n*_2_) and *n*_tot_ = *n*_1_ + *n*_2_. Next, the enantiomeric excess is linked to the newly introduced asymmetry parameter ϕ using the assumption that the shape of the particle size distributions of the two enantiomers is identical, i.e., that they differ only in the number of crystals. In that case, it holds that

9 $$\phi -1=\frac{m_{2,2}}{m_{2,1}}=\frac{n_{2}}{n_{1}}$$

and accordingly that *ee* = ϕ/(2–ϕ). Therefore, the change in the asymmetry during a cycle, Δ*ϕ* = ϕ_*k*+1_–ϕ_*k*_, can be computed as

10 $$\Delta \mathrm{ee}=\frac{\phi_{k+1}}{2-\phi_{k+1}}-\frac{\phi_{k}}{2-\phi_{k}}\approx \frac{\Delta \phi }{2-\phi_{k}}$$

where the approximation holds in the limit of small asymmetries (i.e., for ϕ → 0). Combining eqs 7 and 10 yields an explicit expression for Δ *ϕ*:

11 $$\Delta \phi =\phi_{k}\frac{2\left(2-\phi_{k}\right)K\left(a_{d},\;a_{g},\;\phi \right)}{n_{\mathrm{tot}}}\approx \phi_{k}\mathrm{K̂}\left(a_{d},\;a_{g}\right)$$

It is worth noting that in the limit of small asymmetries, the amplification parameter *K̂* is insensitive to the value of ϕ. This allows formulating a general law for the chiral amplification resulting from *k* subsequent cycles, as to

12 $$\phi_{k+1}=\phi_{k}\left(1+\mathrm{K̂}\left(a_{d},\;a_{g}\right)\right)=\phi_{1}{\left(1+\mathrm{K̂}\left(a_{d},\;a_{g}\right)\right)}^{k}$$

where ϕ_1_ denotes the initial asymmetry of the suspension, which is spontaneously generated as a consequence of the stochastic nature of nucleation. For the simulations shown in Section 3.2, a value of *K̂*(*a*_d_, *a*_g_) = 0.028 is computed (using a crystal growth power of *g* = 1 instead of the value of *g* = 1.05 used in the simulations). This implies that the asymmetry doubles every 25 cycles or every 50 min (given a cycle time of 2 min).

### 5.2 Stochastic Simulations

We simulate a two-phase crystallization process of a chiral compound that forms conglomerates of enantiopure crystals. After an initial cooling phase, during which crystals nucleate and grow, temperature-cycling is carried out to amplify the enantiomeric excess in the solution. We refer to our earlier modeling contributions for detailed discussions on how to consider the stochastic nature of nucleation in simulations of crystallization,^46^ and how to describe temperature-cycling of chiral compounds in a population-based framework,^29^ and discuss in the following the relevant model equations, numerical considerations, and model parameters.

#### 5.2.1 Rate Equations

The behavior of the two enantiomers in solution and suspension is described by two population balances coupled with two material balances, as outlined in Section 5.1. The balance equations are complemented by rate equations for the relevant phenomena, i.e., for primary nucleation, secondary nucleation, crystal growth, and dissolution. Primary and secondary nucleation are described as Poisson processes, so that the number of crystals, *n*, that form in a given time step, *k*, is randomly distributed as follows:

13 $$P_{k}\left(n\right)=\frac{\nu_{k}^{n}}{n!}\;\exp\left(-\nu_{k}\right)$$

whereby ν_*k*_ is the expected number of crystals that nucleate in the time step *k*, given by

14 $$\nu_{k}=\int_{t_{k}}^{t_{k}+\Delta t}K\left(t^{\prime }\right)dt^{\prime }$$

where *K* is the nucleation frequency, i.e., the expected number of nucleation events per unit time, or in other words, the product of the nucleation rate and the volume of the solution. The model considers four such frequencies, namely, one per enantiomer (of which there are two) and one per type of nucleation (primary and secondary). For each enantiomer, the nucleation frequencies are given as

15 $$K_{\mathrm{PN}}=VA_{\mathrm{PN}}S\;\exp\left(-\frac{B_{\mathrm{PN}}}{\ln^{2}S}\right)$$

16 $$K_{\mathrm{SN}}=VB_{\mathrm{SN}}=k_{a}V\mu_{2}k_{\mathrm{SN},a}{\left(S-1\right)}^{s_{a}}$$

It is worth noting that the physicochemical properties of the two enantiomers are identical; hence, their nucleation kinetics are identical as well. Primary nucleation is described using Classical Nucleation Theory (CNT), and secondary nucleation using a power law that scales with the surface area of crystals, as represented by the second moment of the crystal size distribution, μ_2_. Secondary nucleation is assumed to be enantiospecific; i.e., crystals of a certain handedness only nucleate crystals of the same handedness. The quantity *S* is the saturation ratio, defined as *S* = *c*/*c*_eq_(*T*); note that the activity coefficients of the solutes are assumed to change so little in the range of concentrations considered that their ratio, that should appear in this definition, is assumed to be 1.

The rates of crystal growth and dissolution are defined as power laws of the supersaturation as follows:

17 $$G=k_{g}{\left(S-1\right)}^{g}$$

18 $$D=-k_{d}{\left(1-S\right)}^{g}$$

where *k*_d_ = ϕ_d_ *k*_g_. In general, the two exponents might be different, and ϕ_d_ may be any number. In practice, for the sake of simplicity, but without loss of generality, we assume that the two exponents are the same and that ϕ_d_ > 1, in line with our recent contribution.^29^

#### 5.2.2 Solubility Evolution

The two phases of the process are simulated as follows: the initial cooling phase is described by using a linear decrease in solubility over time from a predefined initial value to a predefined lower value in a given time interval. Temperature cycles are also simulated as periodic step changes in the solubility. In both cases, using changes in solubility to simulate temperature changes is possible because the rates of all phenomena in the model are dependent on temperature through supersaturation, hence through solubility only, and not through an explicit temperature dependence. Given that the solubility changes during temperature-cycling are small compared to the initial cooling phase, nucleation is assumed to take place solely during the initial cooling phase but not during temperature-cycling.

#### 5.2.3 Numerical Solution Approach

In the following, we discuss the numerical implementation of the model. Given that nucleation is described as a stepwise Poisson process, simulations are discretized in time with a constant time step Δ*t*. For each time interval *k*, [*t_k_*, *t_k_* + Δ*t*], a discrete number of nuclei *N*_*k*,*i*_ forms at the beginning of the interval for each enantiomer *i. N*_*k*,*i*_ is obtained by drawing a random number from the Poisson distribution. Information on the discrete crystal size distribution *for each enantiomer i* is stored in two time-dependent vectors **L**_*i*_ and **N**_*i*_:

19 $$N_{i}\left(t_{k}\right)=\left[N_{0,i}\left(t_{k}\right),\;N_{1,i}\left(t_{k}\right),\;N_{2,i}\left(t_{k}\right),\;...,\;N_{h,i}\left(t_{k}\right),\;...,\;N_{\mathrm{end},i}\left(t_{k}\right)\right]$$

20 $$L_{i}\left(t_{k}\right)=\left[L_{0,i}\left(t_{k}\right),\;L_{1,i}\left(t_{k}\right),\;L_{2,i}\left(t_{k}\right),\;...,\;L_{h,i}\left(t_{k}\right),\;...,\;L_{\mathrm{end},i}\left(t_{k}\right)\right]$$

**N**_*i*_ denotes the number of nuclei formed at each time step. **L**_*i*_ denotes the sizes of all crystals grouped by the time of their nucleation. This approach relies on the notion that all crystals formed within a time step grow and dissolve in the same manner; hence, the size distribution is fully characterized by two pieces of information, namely the number of crystals formed at each time step and their size. Both vectors depend on time and initially contain only zeroes. The entries in **N**_*i*_ are computed only once due to nucleation, i.e., *N*_h,*i*_, is evaluated at time step *k* = *h*. Before the corresponding time step, they are zero. The entries *L*_*h*,*i*_ in **L**_*i*_ initially are zero, and are updated in every time step *k* ≥ *h* to account for crystal growth (or dissolution in the temperature-cycling phase), as follows:

21 $$L_{h,i}\left(t_{k}\right)=\{\begin{array}{ll} L_{h,i}\left(t_{k-1}\right)+G\left(S_{k,i}\right)\Delta t; & N_{h,i}\left(t_{i}\right)>0\wedge S_{k,i}\geq 1\\ L_{h,i}\left(t_{k-1}\right)+D\left(S_{k,i}\right)\Delta t; & N_{h,i}\left(t_{i}\right)>0\wedge S_{k,i}<1\\ 0; & \mathrm{otherwise} \end{array}$$

where *S*_*k*,*i*_ denotes the supersaturation of the enantiomer *i* at time *t*_*k*_. During dissolution, the set of crystals formed during time step *h* fully dissolves at time step *d*, if *L*_*h*,*i*_ (*t*_*d*_) < 0. Because a negative crystal size is not physically meaningful, we set *L*_*h*,*i*_(*t*_*d*_) to 0 in this case. Further, the number of crystals is set to zero at the given time step and remains so for the remainder of the simulation, i.e., *N*_*h*,*i*_ (*t* ≥ *t*_*d*_) = 0.

The *j*-th moment of the crystal size distribution, μ_j,i_, is defined through the relation

22 $$\mu_{j,i}\left(t_{k}\right)V=L_{i}^{j}\left(t_{k}\right)\cdot N_{i}\left(t_{k}\right)$$

where by the second moment is required to compute the frequency of secondary nucleation as well as the surface ratio ξ and the third moment is used in the mass balance. The concentration *c*_*k*+1,*i*_ for *i* = 1,2 is calculated as

23 $$c_{k+1,i}=c_{k,i}+k_{v}\rho_{c}\left(\mu_{3,i}\left(t_{k-1}\right)-\mu_{3,i}\left(t_{k}\right)\right)+\Delta tk_{r}\left(c_{k,3-i}-c_{k,i}\right)$$

where the subscript 3–*i* = 2 or 1 for *i* = 1 or 2, respectively; hence, it refers to the other enantiomer.

To verify the numerical accuracy of the model, the total mass of solutes in the system, that is, the sum of the mass of the two enantiomers in solution plus the mass of the suspended crystals, was computed at every time step and confirmed to be constant over time. Further, simulations with shorter time steps were carried out and were found not to significantly change the simulation results.

#### 5.2.4 Classification of Primary and Secondary Nuclei

New nuclei may form through either primary or secondary nucleation. In the case of primary nucleation, their handedness is random. In the case of secondary nucleation, the handedness matches that of the parent crystal. In the context of this work, it is useful to determine the number of nuclei that form through each pathway. For this reason, primary and secondary nucleation are considered as two separate Poisson processes, and the number of crystals of each type that nucleate in a time step is determined through a random number drawn from the Poisson distribution. This means that for each time step, a total of four random numbers are drawn: one for each type of nucleation and one for each enantiomer.

#### 5.2.5 Independent Simulations

To characterize the variability of the process, multiple independent simulations were carried out, whereby independence was ensured by using different seeds for random number generation. The model was implemented in MATLAB R2022b in a way that allowed for parallelization, typically employing eight workers. Table 2 provides an overview of all model parameters.

**Table 2:** List of Model Parameters That Were Used in the Simulations Presented in This Work^a^

quantity	abbreviation	unit	value
primary nucleation parameter	*A*_PN_	m^–3^ s^–1^	3.3 × 10^3^
primary nucleation parameter	*B*_PN_		0.127
secondary nucleation parameter	*k*_a_ × *k*_SN,a_	m^–2^ s^–1^	10^9^
secondary nucleation power	*s*_a_		0.98
crystal growth parameter	*k*_g_	m s^–1^	10^–5.4^
crystal growth power	*g*		1.05
dissolution factor	ϕ_d_		6
reaction rate constant	*k*_r_	min^–1^	0.2
solubility (steps at low T)	*c*_eq_	kg m^–3^	20
solubility (steps at high T)	*c*_eq_	kg m^–3^	24
initial solubility	*c*_eq,0_	kg m^–3^	30
initial concentration	*c*_0_	kg m^–3^	30
crystal density	ρ_c_	kg m^–3^	1300
crystal shape factor	*k*_v_		π/4
crystal surface factor	*k*_a_		1
volume	*V*	mL	50
time step	Δ*t*	s	0.5
cooling phase duration	*t*_end_	s	3600
temperature-cycling duration	*t*_end_	h	20
time of growth step	*t*_g_	s	60
time of dissolution step	*t*_d_	s	60

a In the case that specific simulations employed different parameter values, this is highlighted in the corresponding sections.

### 5.3 Deracemization Experiments

#### 5.3.1 Materials and Setup

We deracemized *N*-(2-methylbenzylidene)-phenylglycine amide (NMPA), using a 95/5 (w/w) isopropanol (IPA) and acetonitrile (ACN) mixture as solvent, and the base 1,8-diazabicyclo[5.4.0]­undec-7-en (DBU) as catalyst for the racemization of NMPA. The rate of the racemization reaction as a function of the concentration of NMPA and DBU and of temperature has been studied earlier;^47^ note that the catalyst concentration of 6 μL *g*_s_^–1^ used here corresponds to 31.5 mM in that paper. NMPA was synthesized using the protocol reported earlier.^49^ After sampling, the crystals were washed with *tert*-butyl methyl ether. Chemicals were purchased from Sigma-Aldrich (all with 99% purity) and were used as received.

The experiments were carried out in eight 10 mL cylindrical glass crystallizers (2 cm diameter and 10 cm height) or in a 100 mL glass crystallizer (depending on the volume of the suspension) located in an EasyMax 102 apparatus (Mettler Toledo), which consisted of two identical thermal blocks. One crystallizer per block was equipped with a stainless steel fluorinated ethylene propylene (FEP)-coated thermocouple to monitor temperature. Polytetrafluoroethylene (PTFE) magnetic stirrers were used to stir the suspension at 1000 rpm in the 10 mL crystallizers and at 150 rpm in the 100 mL ones. A stock solution was prepared (100 mL) and distributed to the eight crystallizers (5 mL each) by using preheated single-use syringes.

#### 5.3.2 Experimental Protocol

Experiments consist of four steps. While steps 2 and 3 match the two phases described by the stochastic simulations, step 1 ensures that the solution to be deracemized is indeed racemic. Step 4 describes the mixing of the deracemized suspension and has been carried out only for a subset of experiments.

**1. Preparation of the solution and conditioning**. An undersaturated racemic solution at 60 °C containing the specified amount of the DBU catalyst is prepared. The three different initial concentrations of NMPA, *c*_0_, are bracketed by the NMPA solubilities (as reported earlier^50^) at 40 °C (target temperature after cooling) and at 60 °C, i.e., *c*_eq_(40 °C) = 0.0248 < *c*_0_ = [0.035, 0.048, 0.060] < *c*_eq_(60 °C) = 0.0644; concentrations are given in g solute - the two enantiomers - per g solvent. Two catalyst concentration levels are considered, namely, 6 μL g_s_^–1^ (high catalyst) and 0.6 μL g_s_^–1^ (low catalyst). The solution is left at 60 °C for 2 and 3 h with high and low catalyst concentrations, respectively.

**2. Linear cooling**. The solution is cooled linearly from 60 to 40 °C, at two different cooling rates, *R*_c_= 0.2 °C min^–1^ for slow cooling and 3.3 °C min^–1^ for fast cooling, to trigger nucleation. At the end of cooling, after 40 min of holding time, the suspension is sampled and the enantiomeric excess is measured.

**3. Temperature cycles**. During temperature-cycling, all crystallizers contained DBU at a concentration of 6 μL g_s_^–1^. In experiments with lower catalyst concentration during steps 1 and 2, the remaining amount is added to each crystallizer before starting temperature cycles. Samples are withdrawn and characterized at irregular intervals, and the process is completed when the enantiomeric excess reached a value of 0.98 in all vials. Temperature cycles consist of four phases, as explained elsewhere.^51^ (1) Linear heating from 40 °C to 42.5, 44, and 45.5 °C, for the experiments at *c*_0_ = 0.035, 0.048, and 0.060 gg_s_^–1^, respectively, at a heating rate of 2 °C min^–1^ in all experiments, with the exception of those at the lowest initial concentration of *c*_0_ = 0.035 gg_s_^–1^ where it is 1 °C min^–1^ to avoid temperature overshoot. (2) Holding at a high temperature for 6 min. (3) Linear cooling from the high temperature to 40 °C at a rate of 2 °C min^–1^. (4) Holding at a low temperature for 10 min. The experiments conducted at each of the three initial concentrations vary in their suspension densities and solid-to-liquid mass ratio, upon reaching the target temperature. The temperature levels are set such that for all initial concentrations, a similar fraction of the crystal mass dissolves upon heating.

**4. Mixing suspensions after achieving homochirality**. The eight homochiral suspensions obtained after temperature-cycling are randomly combined in a third vessel, two-by-two, then mixed and subjected to further temperature cycles to deracemize. The resulting four suspensions are again mixed randomly two-by-two and temperature-cycled until homochirality is achieved. Finally, the two resulting suspensions are subjected to the same treatment. When two suspensions with the same handedness are mixed, no temperature cycles are carried out, as the mixed suspension is already homochiral.

#### 5.3.3 Analytics

For monitoring, samples were collected by withdrawing 80 μL of suspension: the first sample was at the end of the cooling phase; the others were taken at irregular intervals during temperature cycles until the suspension reached enantiopurity. The samples were dried by vacuum filtration using a Büchner funnel and an MS PTFE membrane filter with a pore size of 0.45 μm. Crystals were then washed with a few droplets of antisolvent to remove residual amounts of catalyst. Dried crystals were transferred to HPLC vials, dissolved in acetonitrile, and analyzed via HPLC according to the protocol reported earlier.^50^

## Supplementary Material



## Notes

Expansion and analysis of Frank’s model^3^ in the context of our work (PDF)
