# Supplementary material for: Spontaneous Emergence of Homochiral Suspensions from Racemic Solutions via Stochastic Nucleation
Source: J Am Chem Soc. 2025 May 25;147(22):18826–39. doi: 10.1021/jacs.5c02651 (PMC12147123; doi:10.1021/jacs.5c02651)
Supplement: Supplementary file 1 [file ja5c02651_si_001.pdf]

Supporting information for: Spontaneous emergence of homochiral suspensions from racemic solutions via stochastic nucleation

Leif-Thore Deck,[†,‡,¶] Mercedeh Sadat Hosseinalipour,[†,¶] and Marco Mazzotti[*,†]

†*Institute of Energy and Process Engineering, ETH Zurich, 8092 Zurich, Switzerland*

‡*Yusuf Hamied Department of Chemistry, University of Cambridge, Cambridge CB2 1EW, United Kingdom*

¶*L.T.D. and M.S.H. share first authorship of this manuscript and are listed in alphabetical order.*

E-mail: marco.mazzotti@ipe.mavt.ethz.ch

Abstract

This document contains Supporting Information (SI) for the manuscript *Spontaneous emergence of homochiral suspensions from racemic solutions via stochastic nucleation*. It contains an expansion and analysis of Frank's model[1] in the context of our work.

S.1 Frank model

First, we rewrite Equations (9) and (10) in Frank's paper[1], with the additional term accounting for specific antagonism (i.e., $-k_3 n_1 n_2$ as indicated by Frank in his text just before Equation (15)):

$$\frac{\mathrm{d}n_1}{\mathrm{d}t} = n_1(k_1 - k_2 n_1 - (k_2 + k_3) n_2) \tag{S1}$$
$$\frac{\mathrm{d}n_2}{\mathrm{d}t} = n_2(k_1 - k_2 n_2 - (k_2 + k_3) n_1) \tag{S2}$$

Then, introducing a dimensionless time, rescaling concentrations, and combining parameters, i.e., defining $\tau = k_1 t$, $x_i = k_2 n_i / k_1$, and $\delta = k_3 / k_2$, we transform the equations above in the following system of two ordinary differential equations depending on the single parameter δ:

$$\frac{\mathrm{d}x_1}{\mathrm{d}\tau} = x_1(1 - x_1 - (1+\delta) x_2) \tag{S3}$$
$$\frac{\mathrm{d}x_2}{\mathrm{d}\tau} = x_2(1 - x_2 - (1+\delta) x_1) \tag{S4}$$

These equations describe the evolution of the concentrations of the two enantiomers of a chiral substance, (x_1, x_2), in a well-mixed system from an initial state, $(x_{1,0}, x_{2,0})$, where each enantiomer catalyzes its own production (the x_i factor in the right hand side of the $\mathrm{d}x_i/\mathrm{d}\tau$ equation) but with a rate constant that is penalized by its own presence and by the presence of the other enantiomer (the term between brackets in the right hand side of the two equations). The equations can also be viewed as describing in a generic ecosystem the evolution of two species, which compete for the same limited resources. Both species (enantiomers) inhibit the growth (the production) of both, with the parameter δ representing the difference (positive or negative or null) between the effect of one species (enantiomer) on the other species (enantiomer) and the effect on itself. In Frank's paper, δ is positive or zero and represents specific antagonism, but here we broaden the scope of the analysis to include negative values of δ.

If $\delta \neq 0$ the equations above have four equilibria, namely E_1 $(0,0)$, E_2 $(1,0)$, E_3 $(0,1)$, and E_4 $(1/(2+\delta), 1/(2+\delta))$ (see Figures S1(a) and (c). Analyzing trace and determinant of the Jacobian

matrix of the system proves that: E_1 is an unstable node $\forall\delta$; E_2 and E_3 are (unstable) saddle points and asymptotically stable nodes for $\delta < 0$ and $\delta > 0$, respectively; E_4 is an asymptotically stable node and a saddle point for $\delta < 0$ and $\delta > 0$, respectively.

If $\delta = 0$, E_1 is again an unstable node, but the system exhibits also infinite asymptotically stable equilibria along the first quadrant part of the line with equation $x_1 + x_2 = 1$. In this case all trajectories are straight lines of equation $x_2/x_{2,0} = x_1/x_{1,0}$ (see Figure S1(b)).

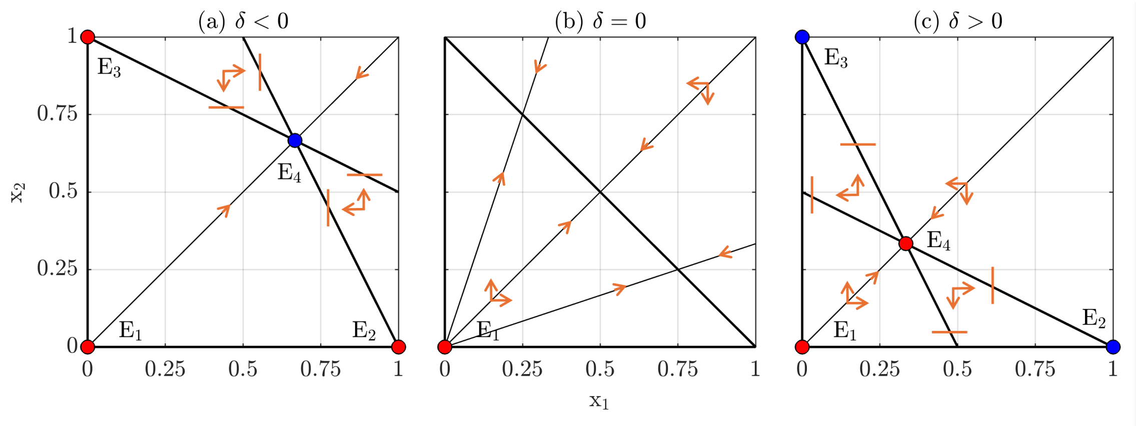

Figure S1: Phase plane representation of the system of ordinary differential equations S3 and S4, with sub-figures (a), (b), and (c) illustrating the cases where $\delta < 0$, $\delta = 0$, and $\delta > 0$, respectively. In all sub-figures blue and red circles are asymptotically stable and unstable (either saddle or unstable node) equilibrium points, respectively; black solid lines are the null-clines of the system of ODEs; thin black lines represent straight line trajectories; little arrows indicate the direction of the vector field generated by the two ODEs. In sub-figure (b), the two null-clines that in the other two cases intersect at the E_4 equilibrium point are the same straight line (with slope -1), hence all the points belonging to this segment are asymptotically stable equilibrium points.

The first quadrant phase plane for two representative cases with $\delta > 0$ (Figure S1(c), corresponding to Frank's case) and $\delta < 0$ (Figure S1(a)) is divided in two parts by the diagonal, which is also the image of two trajectories converging into E_4 hence it cannot be crossed by any other trajectory; it can also be divided in four sectors by the four nullclines, obtained by setting to zero the right hand sides of either equation; the intersections of pairs of the four nullclines are the equilibria of the system, by definition. The features of the vector field associated to the system of ordinary differential equations, which is the set of all tangent vectors to the solution trajectories, whose components are the right hand sides of equations S3 and S4, can be easily assessed (see Figure

S1(a,c)). In the sectors close to the origin and far from it both components of the tangent vectors are positive and negative, respectively. In the two remaining triangular sectors the components of the tangent vectors have opposite signs, and the vector field points towards E_2 and E_3 when $\delta > 0$ and towards E_4 when $\delta < 0$, which is obviously consistent with the stability analysis above.

It is also worth noting that in any given time interval $\Delta\tau$ the amplification of the asymmetry, i.e., of the difference $x_1 - x_2$ (assuming $x_1 > x_2$ at a specific time during the evolution of the system), is obtained by subtracting equations S3 and S4 term by term:

$$\Delta(x_1 - x_2) = (x_1 - x_2)(1 - x_1 - x_2)\Delta\tau \tag{S5}$$

Let us consider the sign of this quantity in a neighborhood of the racemic equilibrium E_4 only, because it is the stability of E_4 that decides whether the ultimate state of the system is racemic or homochiral. In the case $\delta > 0$ the quantity $\Delta(x_1 - x_2)$ is positive because E_4 is located below the line $x_1 + x_2 = 1$ hence $1 - x_1 - x_2 > 0$ and the asymmetry is amplified, whilst in the case $\delta < 0$ this quantity is negative because E_4 is located above that line and the asymmetry is reduced.

Thus summarizing, if $\delta > 0$ and inhibition from the other species (enantiomer) is stronger than inhibition from itself, then co-existence is impossible and the ultimate state is a single-species (homochiral) state, whereby any initial asymmetry, though small, is amplified. If $x_{1,0} > x_{2,0} > 0$ the ultimate state will asymptotically be E_2; if the opposite is true than it will be E_3. In other words, the majority species (enantiomer) at the start will be the only surviving species (enantiomer) at the end. If on the contrary $\delta < 0$ and inhibition from the other species (enantiomer) is weaker than inhibition from itself, then co-existence is the only stable state and any initial asymmetry, though large, is ultimately symmetrized (the ultimate state will asymptotically be E_4). If $\delta = 0$ and the system exhibits unspecific antagonism, then "any initial disproportion is preserved, but not amplified."[1] It is worth underlining that these conclusions just expand Frank's results to the case where $k_3 < 0$, using a simplified model depending on a single parameter, i.e., δ: both extensions are worthwhile for the purpose of the comparison with solid-state deracemization.

References

(1) Frank, F. On spontaneous asymmetric synthesis. *Biochimica et Biophysica Acta* **1953**, *11*, 459–463, DOI: 10.1016/0006-3002(53)90082-1.
